# Isolated Splenic Tuberculosis without Any Radiological Focal Lesion

**DOI:** 10.1155/2015/130209

**Published:** 2015-01-06

**Authors:** Sunil Raviraj, A. Gogia, A. Kakar, S. P. Byotra

**Affiliations:** Department of Medicine, Ganga Ram Institute of Postgraduate Medical Education and Research Center (GRIPMER), Sir Ganga Ram Hospital, New Delhi 110060, India

## Abstract

Incidence of tuberculosis (TB) of spleen is a rare entity and isolated splenic tuberculosis is an unusual phenomenon, especially in immunocompetent individuals. We came across a case of 63-year-old male who presented with high grade fever, loss of weight, and generalized weakness of one-month duration. When physically examined, he had pallor and moderate nontender splenomegaly without any other significant clinical findings. He had pancytopenia, elevated ESR, and positive Mantoux test. Ultrasonography and CT scan of abdomen showed splenomegaly without any other relevant findings. Markers of connective tissue disorders and bone marrow aspiration and biopsy all were noncontributory for diagnosis. Hence splenic biopsy was done and sent for histopathological examination. Presence of caseation surrounded by epitheloid granulomas and Langerhans cells was suggestive of diagnosis as tuberculosis. And gene probe for the AFB (acid fast bacilli) came to be positive. No primary focus was present in either lungs or other organs. Patient improved clinically with antitubercular treatment.

## 1. Introduction

Tuberculosis is a bacterial infection which is caused by* Mycobacterium tuberculosis*. In spite of early diagnostic and extensive treatment modalities the incidence of disease is still the major public health problem in developing countries [1]. While pulmonary tuberculosis is the most common manifestation, extra pulmonary disease accounts for approximately 15% of all tuberculosis [2]. Splenic tuberculosis is exceptionally rare entity which usually occurs as a part of miliary tuberculosis and is more common in immunodeficient individuals. Here we are reporting a case of splenic TB, as it is a rare entity.

## 2. Case Report

**Figure 1:**
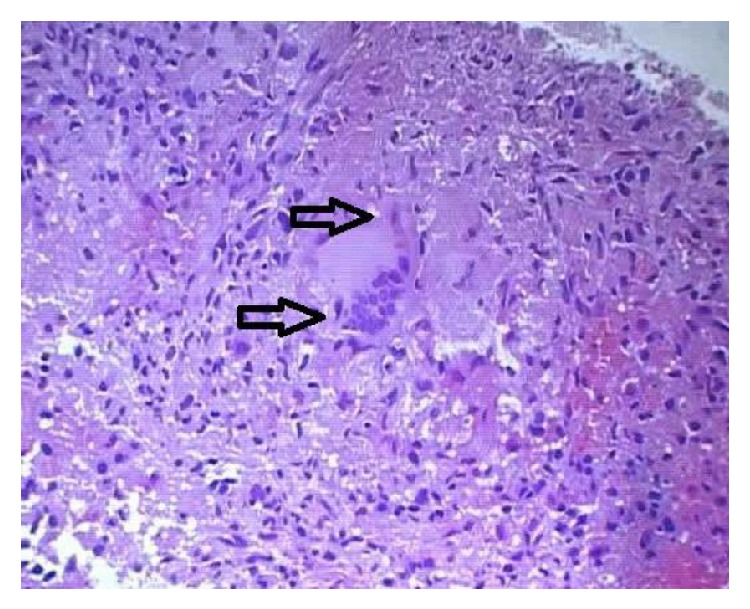
Histopathological examination of splenic tissue showing epithelioid cells and Langerhans cell histiocytes, suggestive of tuberculosis.

A 63-year-old male known diabetic (in control with oral hypoglycemic agents), from Jharkhand (eastern state of India), presented with complaints of high grade fever associated with chills and rigor along with loss of weight and loss of appetite of one-month duration. There was no history of cough, breathlessness, chest pain, pain abdomen, or diarrhea. On clinical examination, he had pallor and moderate nontender splenomegaly without any other significant findings. With provisional diagnosis of malaria, antimalarials had been started along with broad spectrum antibiotics and other symptomatic treatments. His hemogram revealed pancytopenia (hemoglobin: 8.7 gm%, total leucocyte count: 3200 cells/cumm, and platelets: 1,09,000/cumm). His erythrocyte sedimentation rate was elevated markedly (90 mm in 1st hour). Peripheral smear was consistent with normocytic normochromic picture without any abnormal cells. Liver and renal function tests were within normal limits (total bilirubin: 0.9 mg/dL, direct bilirubin: 0.3 mg/dL, total protein: 6.9 gm/dL, albumin: 3.2 gm/dL, SGOT/SGPT: 60/54 IU/L, and ALP/GGT: 100/110). Fasting and postprandial blood sugar were 123 mg/dL and 189 mg/dL, respectively. His HbA1c was 7.4. Chest X-ray was unremarkable. Ultrasound (USG) abdomen was done which showed gross splenomegaly without any focal lesion. Contrast enhanced CT scan (CECT) thorax and abdomen confirmed the USG findings and did not show any other abnormalities. Blood and urine cultures were negative. Hepatitis B, hepatitis C, and HIV markers were negative. As fever, splenomegaly, and pancytopenia were persisting, possibility of Kala-Azar was suspected, which is ramped in eastern India [3]. Workup for leishmaniasis was done. Bone marrow aspiration and biopsy were negative for* Leishmania* bodies and there were no abnormal cells apart from few hemophagocytes. Simultaneously antibody to K39 antigen (*Leishmania* antibody) was sent and result was negative. Markers of connective tissue disorders (anti-nuclear antibody, ENA profile, c-ANCA, and p-ANCA) were negative. Empirically antifungal agents were started. In view of persisting fever despite broad spectrum antibiotics, splenic biopsy was done under ultrasound guidance and sent for histopathological examination. The caseating necrotic areas along with epithelioid granulomas and Langerhans cells in biopsy tissue were suggestive of tuberculosis (Figure 1). Gene probe for AFB was positive but stain for acid fast bacilli was negative. Therefore, final diagnosis of isolated splenic tuberculosis was made as there was no focus in either lungs, gastrointestinal tract, or lymph node. Thus, the patient was started on antitubercular treatment after which his fever subsided and his general condition improved.

## 3. Discussion 

Tuberculosis is common infection caused by various strains of mycobacteria, most commonly by* Mycobacterium tuberculosis*. Splenic TB can occur in two forms: one is as a part of disseminated disease and second is isolated form. Spleen is the third most common organ (lung 100%, liver 82%, spleen 75%, lymph nodes 55%, and bone marrow 41%) [4] involved in miliary tuberculosis, but isolated splenic tuberculosis is a very rare entity. Most of the times splenic TB presents as fever of unknown origin (FUO) [4] as in our patient. In immunocompromised states like HIV/AIDS, splenic involvement is common as a part of disseminated tuberculosis. Although our patient had no major immunocompromised state, being a diabetic he was at risk of developing tuberculosis [5]. A study done by Alavi et al. showed high prevalence of tuberculosis and smear positivity rate in diabetes people compared to in nondiabetics [6]. Diagnosis of isolated splenic TB is difficult and is often delayed because of imprecise clinical manifestations. Radiological findings are the key for getting diagnosis in majority of cases, even though histological and/or microbiological evidence is the confirmatory. Most typically, splenic lesions present as multiple, regular hypoechoic nodules on ultrasound (USG), which represent tuberculomas or abscess [6]. Usually CT scan does show hypo- or hyperdensities in splenic TB, but in most of the instances it is misdiagnosed as lymphoma or fungal abscess. In a case series reported by Dalal et al., [7] five of the six patients with findings of isolated splenomegaly on USG were found to have lesions on CT scan, whereas our patient had no focal lesion either in USG or in CT scan. At this stage our differential diagnoses were tuberculosis, Kala-Azar, and lymphoma. For definitive diagnosis tissue biopsy was planned and splenic biopsy under ultrasound guidance was done. Multiple caseating necrotic regions surrounded by epithelioid granulomas and Langerhans giant cells in biopsy were suggestive of tuberculosis and gene probe for AFB also came to be positive. Histopathological and/or microbiological evidence is a must to diagnose splenic tuberculosis. In a report of solitary splenic TB by Fooladi et al., microbiological and molecular examinations were carried out which are helpful in etiological diagnosis [8]. Antitubercular treatment is the primary modality in treating splenic TB. Splenectomy can be required in the presence of splenic abscess or splenic rupture.
